# Electrodeficient Diborane (4) Converted into an Extraordinary Proton Sponge

**DOI:** 10.1021/acs.jpca.5c08104

**Published:** 2026-01-29

**Authors:** Manuel Yáñez, M. Merced Montero-Campillo, Otilia Mó, Ibon Alkorta

**Affiliations:** † Departamento de Química, Módulo 13, Facultad de Ciencias, and Institute for Advanced Research in Chemical Sciences (IAdChem), Universidad Autónoma de Madrid,Campus de Excelencia UAM-CSIC, Cantoblanco,Madrid 28049, Spain; ‡ Instituto de Química Médica, IQM-CSIC, Juan de la Cierva, 3. Madrid 28006, Spain

## Abstract

High-level G4 calculations show that the interaction of diborane(4) (B_2_H_4_) with nitrogen bases not only stabilizes the *C*_*2v*_ isomer with respect to the *D*_*2d*_ one, but more importantly retains and enhances the distinctive reactivity of the *C*_*2v*_ isomer. The formation of the complex results in a large enhancement of the donor ability of the diborane subunit. As a first consequence, the boron site is by far more basic than nitrogen in terms of enthalpy, leading to protonated complexes that can be viewed as the association of the different bases to the B_2_H_5_^+^ cation. Further analysis of the electron density redistribution upon complexation helps to rationalize the key factors behind the drastic basicity enhancement observed. The basicity of the B_2_H_4_–pyridine complex falls within the range of gas-phase superbases, with a calculated proton affinity (PA) exceeding 1000 kJ·mol^–1^. Moreover, complexes with stronger bases, such as guanidine and methyl-substituted imidazoles, surpass the basicity of the prototypical proton sponge and superbase 1,8-bis­(dimethylamino)­naphthalene. Precisely, B_2_H_4_–1,2,5-trimethylimidazole is predicted to be a boron base 34 kJ·mol^–1^ more basic than the proton sponge, corresponding to an increase in the protonation equilibrium constant of nearly 6 orders of magnitude.

## Graphical abstract

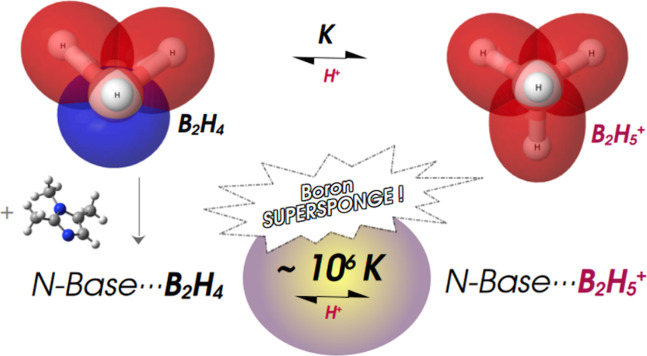

## Introduction

Electron-deficient systems, in particular triel and alkaline-earth derivatives, are prototypical Lewis acids and most of their chemistry is dictated by this characteristic.^1,2^ These Lewis acids, when interacting with conventional Lewis bases, yield complexes stabilized by rather strong dative bonds, formed by the transfer of the Lewis base lone pair into the appropriate empty orbital of the Lewis acid. The immediate consequence is a drastic reorganization of the Lewis base reflected in a significant acidity enhancement of the base. First evidence of this behavior was reported in 2009 in a combined experimental-theoretical study on the enhanced acidity of phosphines when associated with borane.^3^ This work was followed by a series of studies exploring a wide range of Lewis pairs, further substantiating and expanding the initial findings.^4–8^

Although all dative-bond interactions of this kind are expected to induce these effects in the acidity of the Lewis base, relatively little attention has been given to a possible enhancement of the basicity of the Lewis acid. Parallel increases in both acidity and basicity have been reported for water when interacting with various Lewis acids and bases.^9^ Similarly, the intrinsic basicity of XH_n_OH compounds increases significantly through noncovalent interactions with Lewis bases.^10^ Nevertheless, much less is known about highly electrodeficient Lewis acids behaving like bases thanks to strong interactions with donors. This scarcity may stem from the difficulty of envisaging such species as likely to donate electrons. Furthermore, some Lewis acids, such as boranes or Be and Mg hydrides, possess hydrogen atoms carrying a partial negative charge, whose confrontation with nitrogen bases might lead to typical hydride abstraction reactions. This mirrors recently findings for complexes of BeX_2_ /MgX_2_ (X = H, F) with NH_3_, CH_2_NH, HCN, and NC_5_H_5_.^11^ In this case, protonation occurs at the negatively charged hydrogen atoms of the alkaline hydrides, while protonation at the N atom of the base is systematically less exothermic.^11^

In contrast to previous work, which focused on the dramatic enhancement of acidity, here we demonstrate that association with N-bases can also transform B_2_H_4_ into a boron-centered superbase whose PA surpasses that of the prototypical proton sponge.

The previously mentioned findings in alkaline-earth hydrides prompted us to explore whether a similar behavior would occur when the Lewis acid is a boron-containing derivative. We selected diborane(4) for this investigation, as its interaction with nitrogen bases is known to enhance their acidity by 38–58 orders of magnitude relative to the corresponding free bases, effectively transforming them from varying levels of basicity into superacidic species.^12^ For this purpose, we have started investigating the basicity of complexes between B_2_H_4_ and the N-bases included in ref.,^12^ namely ammonia, ethylenimine, hydrogen cyanide, and pyridine. In view of the results obtained, we decided to enlarge the set by including stronger N-bases, namely, guanidine and methylated imidazoles (1-Me-, 2-Me-, 1,2-diMe-, 2,4-diMe, 1,2,4-triMe, and 1,2,5-triMe-imidazole), to explore if larger proton affinities for diborane(4) could be obtained.

## Computational Details

In order to guarantee the desired reliability for our predictions, we have used the high-level G4 ab initio composite method.^13^ The accuracy of G4 was benchmarked against the G3/05 test set,^14^ yielding an average absolute deviation of 3.5 kJ·mol^–1^ from experiment.^13^ However, this approach may fail if the system is governed by dispersion forces,^15^ as it is based on B3LYP optimized geometries. To verify that this is not the case for the systems under investigation, the G4 geometries were checked against M06–2X/aug-cc-pVTZ geometries, a functional that correctly accounts for dispersion contributions. All these calculations have been carried out with the Gaussian-16 program.^16^ Unless otherwise stated, all proton affinities and complexation enthalpies correspond to G4 electronic energies plus G4 thermal corrections at 298 K.

The bonding characteristics of the B_2_H_4_–N-base complexes were analyzed using the adaptive natural density partitioning (AdNDP) approach,^17^ within the general framework of the natural bond orbital (NBO) procedure.^18^ This technique is particularly well-suited to describe (3c,2e) bonds, which play a crucial role in stabilizing the most stable *C*_*2v*_ structure of B_2_H_4_ and that of its most stable B_2_H_5_^+^ protonated species. Complementary information was gathered through a topological analysis of the electron density by means of the quantum theory of atoms in molecules (QTAIM),^19^ which provides the complete set of electron density critical points defining the corresponding molecular graph, and by the use of the electron localization function (ELF) approach,^20^ which allows us to locate and classify basins associated with core electrons and lone pairs (monosynaptic), two-center bonds (disynaptic) and n-center bonds, with *n* ≥ 3 (polysynaptic). All bonding and electron density analysis have been carried out at the M06–2X/aug-cc-pVTZ level of theory.

As a binary complex arises from the interaction between two monomers, there is a distortion of electron densities with respect to the isolated structures. The information provided by the one- and two-body contributions to the binding energy helps to understand the stability trends of the neutral and protonated complexes. This can be easily done in the framework of the many-body interaction-energy (MBIE) formalism.^21,22^ The MBIE approach defines one- and two-center terms for an AB complex as shown in eqs 1 and 2:

$$E_{R}(A)=E_{m}(A)-E(A)$$ 1

$$\Delta^{2}E(AB)=E(AB)-[E_{m}(A)+E_{m}(B)]$$ 2

where *E*(*A*), *E*(*B*) and *E*(*AB*) represent the energies of monomers *A* and *B*, and the *AB*-dimer, respectively, at their equilibrium geometries. *E*_m_(*A*) and *E*_m_(*B*) denote their energies at the geometry they adopt within the *AB* complex. Accordingly, the total interaction energy will be given by eq 3:

$$\Delta E=E\left(AB\right)-E\left(A\right)-E\left(B\right)=E_{R}\left(A\right)+E_{R}\left(B\right)+\Delta^{2}E\left(A,B\right)$$ 3

## Results and Discussion

As a preface to the investigation of the complexes, it is worth recalling some key milestones in the study of diborane(4). Early investigations of this compound considered exclusively *D*_*2d*_ and *D*_*2h*_ conformations^23–28^ ; later, MP2/6-31G* calculations revealed the existence of a doubly bridged *C*_*2v*_ structure slightly less stable than the *D*_*2d*_ form.^29^ Three years later, Curtiss and Pople revisited the stability of the *C*_*2v*_ isomer at the G1 level of theory, finding it to be marginally more stable than the *D*_*2d*_ conformer.^30^ In the same year, photoionization mass spectrometry provided experimental evidence for a doubly bridged *C*_*2v*_ geometry. Our recent G4 calculations predict the *C*_*2v*_ B_2_H_4_ to be 5.2 kJ·mol^–1^ more stable than the *D*_*2d*_ conformer. This energy gap decreases slightly in terms of Gibbs free energy but, even at room temperature, approximately 76% of B_2_H_4_ exists in the *C*_*2v*_ conformation at equilibrium.

The fact that B_2_H_4_ adopts a doubly bridged *C*_*2v*_ structure is far from trivial, as it is associated with a peculiar bonding arrangement involving a high electron density in and around the B–B internuclear region.^31^ Our M06−2X/aug-cc-pVTZ calculations reproduce well the geometry previously obtained at the MP2 level.^29,31^

As shown in Figure 1a, the molecular electrostatic potential (MEP) reveals a pronounced nucleophilic region below the B–B region, consistent with the AdNDP description of two (3c,2e) B–H–B bonds plus a B–B bonding orbital in Figure 1b. In Figure 1c, the ELF analysis finds a polysynaptic basin populated by 2.02 e, shaped like a typical heteroatom lone pair. The QTAIM description in Figure 1d reveals a peculiar connectivity pattern, with the presence of a non-nuclear attractor (NNA) with a notably high electron density. Not surprisingly, B_2_H_4_ behaves as a nonclassical electron donor, readily forming highly stable complexes upon interaction with various hydrogen-bond donors.^31^ These features are consistent with protonation of B_2_H_4_ yielding a *C*_*3v*_ B_2_H_5_^+^ cation,^32,33^ stabilized by the formation of a new (3c,2e) B–H–B bond (see Figure 2). Interestingly, this electron-deficient compound is predicted to be unexpectedly basic, as its G4-calculated proton affinity (816.9 kJ·mol^–1^) exceeds that of typical bases such as phosphine (785.0 kJ·mol^–1^).^34^

**Figure 1:**
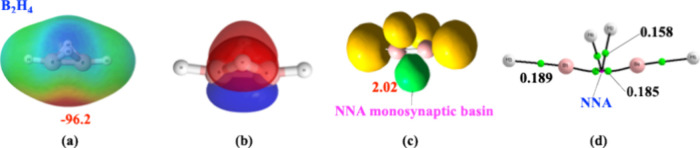
Different chemical descriptions of the B_2_H_4_ molecule. (a) Molecular electrostatic potential. The red area is the nucleophilic region, the energy of the global maximum in kJ·mol^–1^. (b) Molecular orbitals provided by the AdNDP description: in red, two (3c,2e) B–H–B bonds, and in blue, the B–B orbital. (c) Electron localization function representation. In yellow, disynaptic and trisynaptic basins involving H atoms. In green, a monosynaptic basin associated with the non-nuclear attractor (NNA) shown in (d). Population in e. (d) Molecular graph. Green dots are bond critical points; the red point is the NNA. Electron densities in a.u.

**Figure 2:**
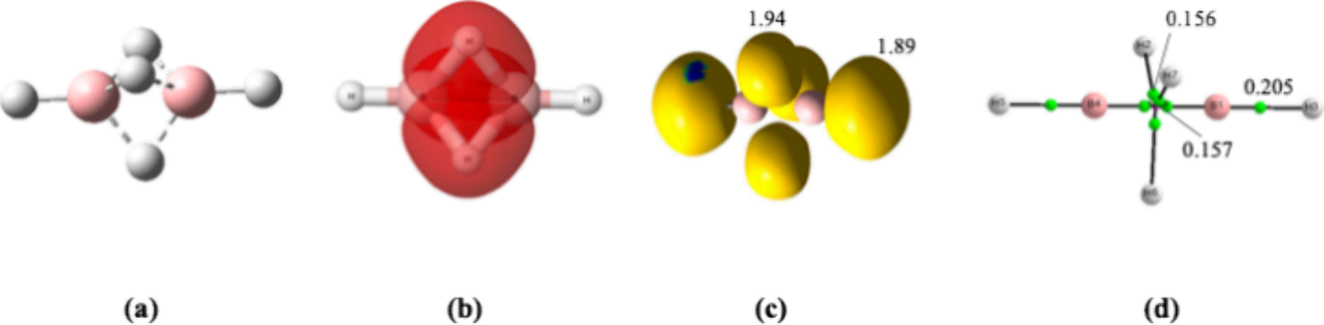
(a) Structure of B_2_H_5_^+^. (b) Molecular orbitals provided by the AdNDP description, showing the three (3c,2e) B–H–B bonds. (c) The electron localization function representation, showing the disynaptic and trisynaptic basins involving H atoms and their populations. (d) Molecule graph. Same conventions as in Figure 1.

With this background in mind, our objective is to determine whether the interaction with N-bases enhances the intrinsic basicity of B_2_H_4_. In this context, the first key finding is that association with various N-bases increases the stability of the *C*_*2v*_ isomer relative to the *D*_*2d*_ isomer. For sufficiently strong N-bases such as pyridine, only the complex derived from the *C*_*2v*_ isomer remains stable.^12^ More importantly, the distinctive B–B bonding characteristics of B_2_H_4_ are retained in the B_2_H_4_–N-base complexes, as shown in Figure 3 using the complexes with ammonia and guanidine as examples of moderate and strong bases, respectively. Although formation of the N → B dative bond leads to the loss of one of the (3c,2e) B–H–B bonds, the B–B bonding orbital is preserved, exhibiting a comparable population at the related ELF basin (2.01 e for the ammonia complex and 1.99 e for the guanidine complex vs to 2.02 e for the parent B_2_H_4_ compound). This basin, however, undergoes a pronounced reduction in volume when compared to the B_2_H_4_ one shown in Figure 1c, going from 126.39 u^3^ to 83.99 u^3^ and 80.57 u^3^ in the ammonia and guanidine complexes. Notably, complexation leads to loss of one B–H–B 3c–2e bond but retention of a compact B–B basin as the primary electron donor. The contraction of both donor basins is reflected in the disappearance of the NNA found in the isolated boron species, now replaced by a B–B bond critical point, together with a very severe increase in the depth of the molecular electrostatic potential (MEP).

**Figure 3:**
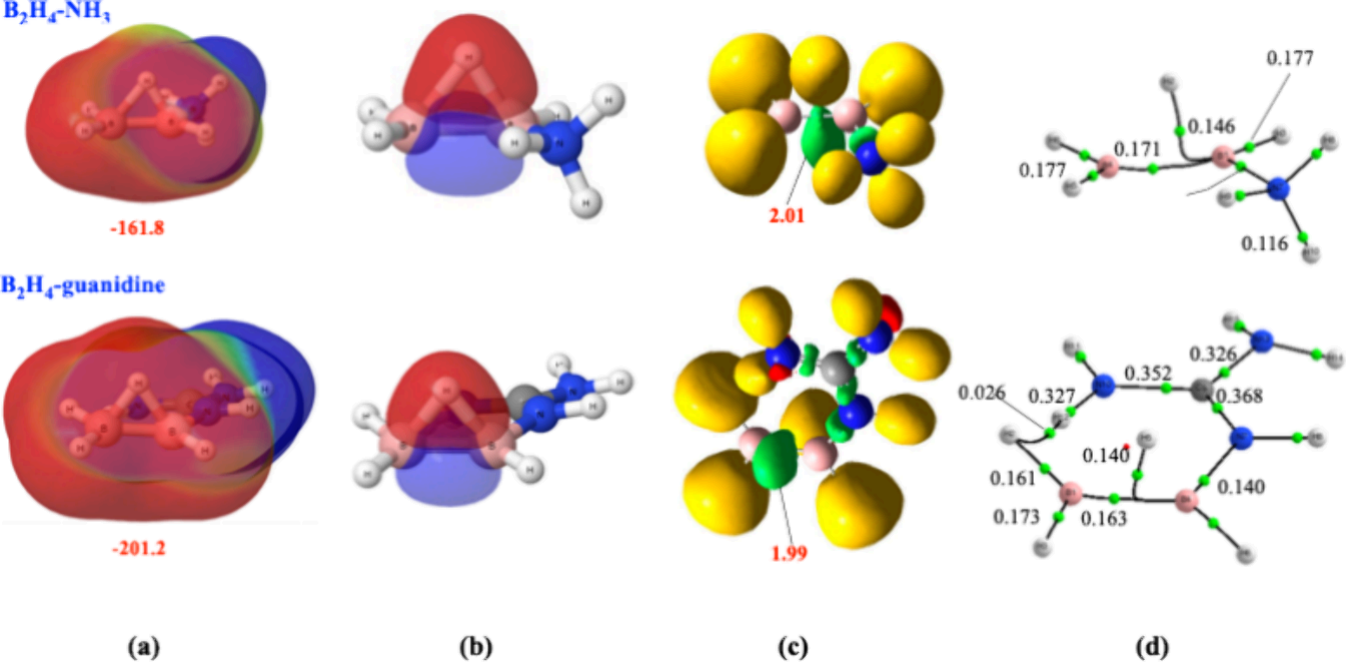
(a) Molecular electrostatic potential, (b) AdNDP description, (c) electron localization function representation, and (d) molecular graph for the B_2_H_4_–NH_3_ and B_2_H_4_–guanidine complexes. Same conventions as in Figure 1.

However, the topology of the MEP in the complexes suggests that protonation may occur not only at the B–B bond, but also at hydrogen atoms bound to boron or at the N-base. Although possible, the most favorable process is protonation at the B–B bond, as illustrated in Figure 4, using the B_2_H_4_–NH_3_ complex as an example. The fact that N-base is less basic allows the protonated species to be regarded as the result of the association of the N-base to the B_2_H_5_^+^ cation in almost all cases.

**Figure 4:**
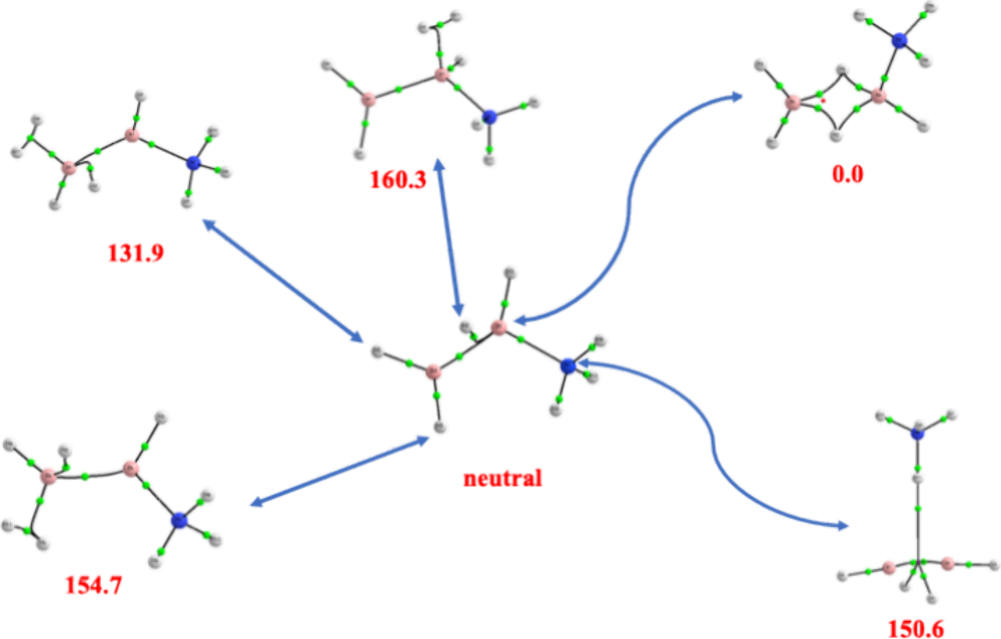
Protonated forms of the B_2_H_4_–NH_3_ complexes for different protonation sites. Relative proton affinities in kJ·mol^–1^.

It is interesting to note that protonation at negatively charged hydrogens leads to the formation of a hydrogen molecule in three of the protonated complexes (+131.9, +154.7, +160.3 kJ·mol^–1^); one of these species is even more stable than the N-protonated complex (+150.6 kJ·mol^–1^). The predominance of the hydride abstraction over protonation at the N-base has been observed for complexes involving the monomers and dimers of BeH_2_ and MgH_2_,^11^ owing to the high exothermicity of the reaction H^+^ + H^–^ → H_2_ (see Figures S1–S4 of the Supporting Information and the corresponding discussion). In the case of neutral diborane(4), the acidity of these hydrogens could have been anticipated from the maxima of the MEP (see Figure S5 of the Supporting Information).

As pointed out before (Figure 3), the higher MEP values of the complexes with respect to that of diborane(4) made us expect a boron basicity enhancement for this compound upon interaction with N-bases. This prediction holds true for all complexes investigated, as summarized by the calculated PAs in Table 1. A first examination comprised only the first four complexes, which already demonstrated that the B_2_H_4_–pyridine complex behaves as a superbase, with a PA exceeding 1000 kJ·mol^–1^. Looking for stronger N-bases that could help to reach the basicity of the proton sponge 1,8-bis­(dimethylamino)­naphthalene (experimental PA 1028.2 kJ·mol^–1^),^35^ we extended the survey to five additional complexes listed in Table 1. As anticipated in the Introduction, our expectation was confirmed: the new complexes, involving guanidine and the selected methylated imidazole family, are indeed stronger bases than the proton sponge. Even more remarkably, while the proton sponge is a nitrogen base, the B_2_H_4_–N-base complexes behave as boron bases!

**Table 1:** G4 Proton Affinities (PA) of the B_2_H_4_–N-Base Complexes after Protonation at the B_2_H_4_ Moiety^a^

system	proton affinity (PA)	ΔPA
B_2_H_4_	816.9	0.0
B_2_H_4_–NH_3_	961.0	144.1
B_2_H_4_–NH=CH_2_	968.6	151.7
B_2_H_4_–NCH	936.3	119.4
B_2_H_4_–pyridine	1006.0	189.1
B_2_H_4_–guanidine	1042.5	225.6
B_2_H_4_–1-Me imidazole	1044.5	227.6
B_2_H_4_–2-Me imidazole	1042.9	226.0
B_2_H_4_–1,2-diMe imidazole	1052.9	236.0
B_2_H_4_–1,2,5-triMe imidazole	1062.2	245.3

a ΔPA represents the increase in PA of the complex relative to that of the isolated B_2_H_4_ molecule. All values are in kJ·mol^–1^.

It is tempting to infer that the greater the basicity of the N-base, the larger the basicity enhancement in the B_2_H_4_–N-base complexes. In fact, the linear correlation plotted in Figure 5 indicates a good agreement between the basicity of the N-base and the basicity enhancement in the complex, although the regression coefficient is slightly below 0.9 (*R* ^2^ = 0.8939).

**Figure 5:**
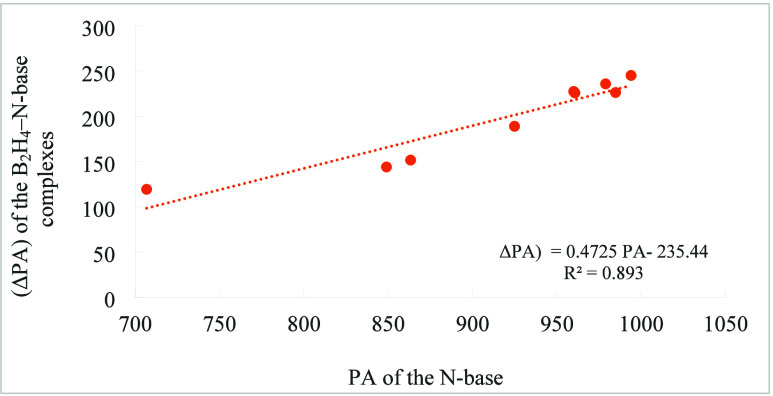
Linear correlation between the G4-calculated basicity enhancement (ΔPA) of the B_2_H_4_–N-base complexes protonated at the B_2_H_4_ moiety and the proton affinity (PA) of the corresponding N-base. All values in kJ·mol^–1^.

Looking at the results in Table 1, it is not straightforward to understand why guanidine, a stronger base than 1-methylimidazole (PA = 984.7 vs 959.9 kJ·mol^–1^), yields a basicity enhancement almost identical and even slightly lower than that for the 1-methylimidazole complex (225.6 kJ·mol^–1^ vs 227.6 kJ·mol^–1^). The reason behind this result is the tight relationship between the basicity enhancement ΔPA and the complexation reactions for both the neutral and protonated species.^3^ This is reflected in a thermodynamic cycle in Figure 6, from which it is possible to derive eq 4:

$$\mathrm{\Delta PA}=\mathrm{PA}-{\mathrm{PA}}^{0}={\mathrm{\Delta H}}_{r\;p}-{\mathrm{\Delta H}}_{r\;n}$$ 4

**Figure 6:**
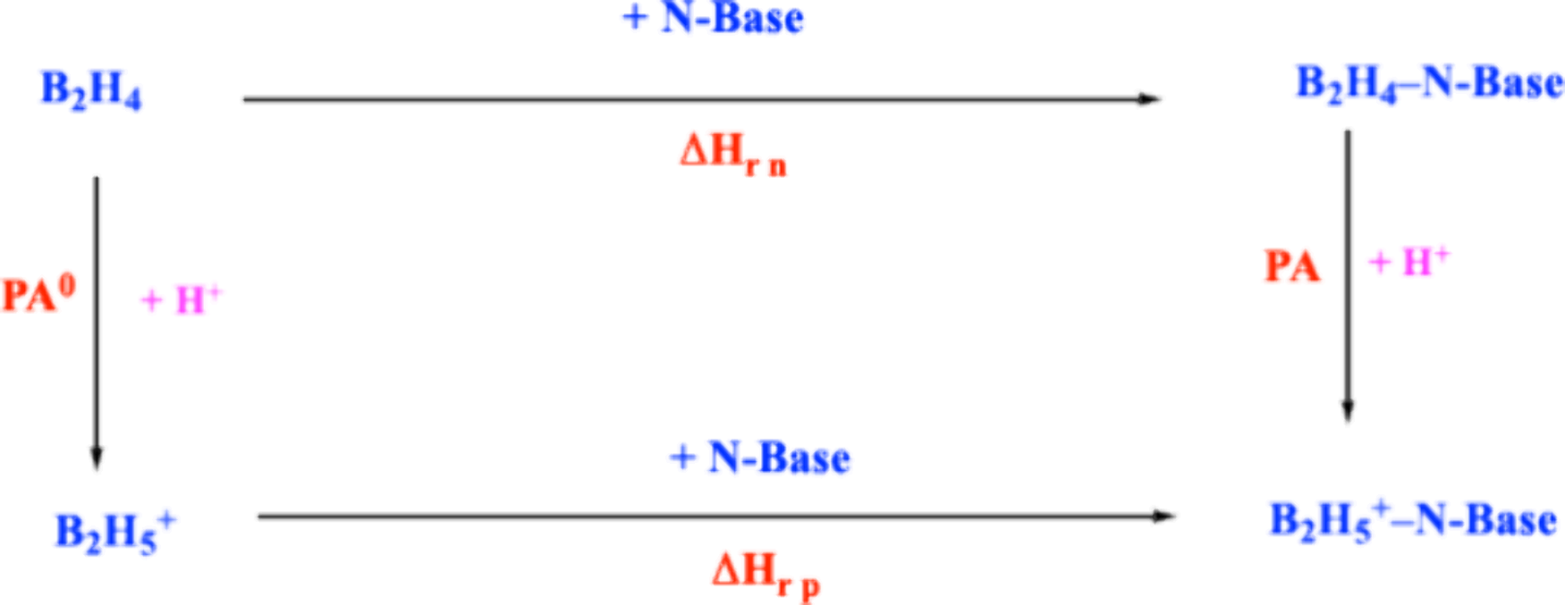
Thermodynamic cycle showing the relationship between basicity enhancement and complexation enthalpies for neutral and protonated species.

It is clear, therefore, that the increase in basicity is proportional to the difference in complexation between diborane(4) and the N-base. In principle, a better base should guarantee a stronger interaction upon complexation, but only if the geometry of the complex involves negligible or little distortion from the original isolated moieties. Moreover, if more than one arrangement is possible, we cannot ensure that the most stable arrangement for the neutral species is still the same upon protonation. The B_2_H_4_–guanidine complex provides a clear example of this effect. As illustrated in Figure 7, the neutral complex exhibits two conformers **A** and **B.** Thanks to a stronger intramolecular dihydrogen bond, **A** is 12.9 kJ·mol^–1^ more stable than **B**, which contains a weaker interaction of the same kind. The stability order reverses in the protonated forms, where **B** becomes the most stable.

**Figure 7:**
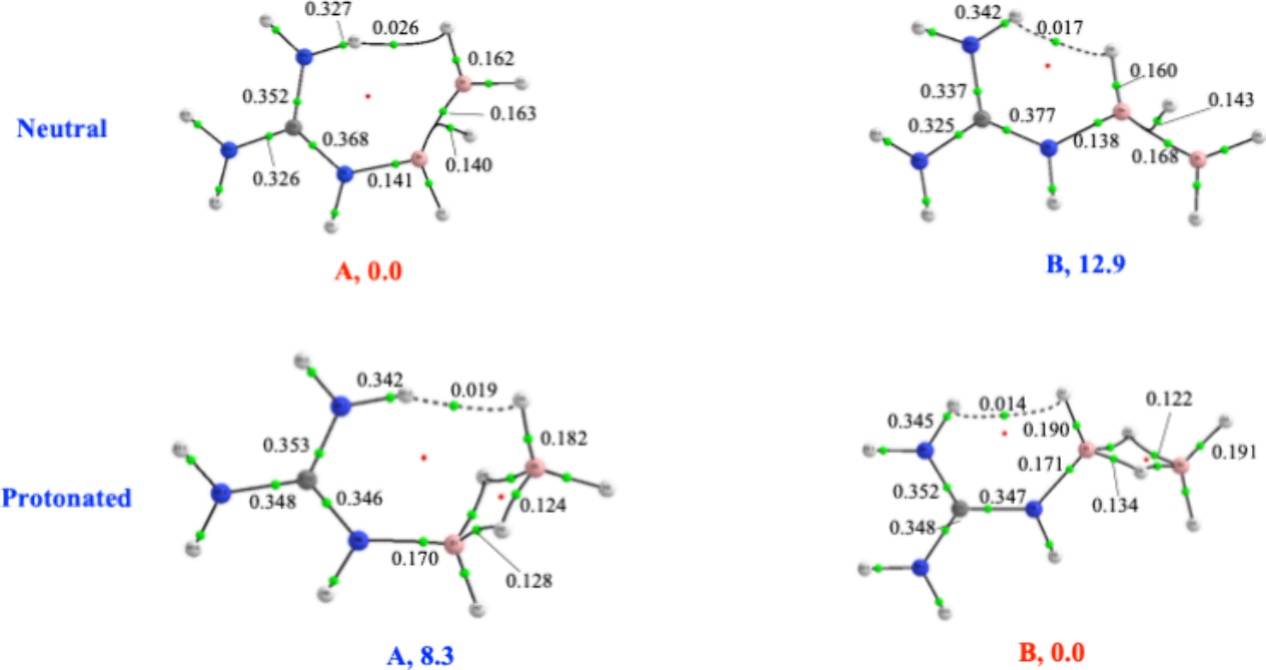
Molecular graph for the two conformers **A** and **B** of the B_2_H_4_–guanidine complex and their protonated species. Relative stabilities are in kJ·mol ^–1^.

It is possible to evaluate the strength of the interaction and the distortion penalty with the MBIE method described in the Computational Details section for different conformers of the same system. Table 2 compares, for instance, the deformation energies of the monomers (positive E_R_ values) and the stabilizing two-center term (Δ^2^ *E­(AB)* for the neutral and protonated B_2_H_4_–guanidine complexes. It should be noted that MBIE calculations are performed using energies rather than enthalpies; therefore, values in Table 2 differ slightly from those in Table 1, which include enthalpy thermal corrections. For the neutral complex, the Δ^2^ *E*(*AB*) term is ca. 38 in kJ·mol^–1^ stronger for conformer **A**. However, conformer **A** also exhibits larger monomer distortion penalties—about 26 kJ·mol^–1^ higher than in conformer **B**—so its overall stability relative to **B** is reduced to roughly 12 kJ·mol^–1^. Upon protonation, the situation remains qualitatively similar but changes quantitatively. For the protonated complexes, the variation of the Δ^2^ *E*(*AB*) term decreases from ca. 38 to ca. 5 kJ·mol^–1^ when going from conformer **A** to **B**, while the change in the overall monomer deformation energy decreases from ca. 26 to ca. 16 kJ·mol^–1^. As a result, the protonated conformer **B** becomes now the global minimum.

**Table 2:** G4 MBIE Results for the B_2_H_4_–N-Base and [B_2_H_5_–N-Base]^+^ (N-Base = Guanidine, 1-Methyl-imidazole) Complexes, Formed by Attaching the N-Base to the Most Stable Conformers of B_2_H_4_ (*C*_*2v*_) and B_2_H_5_^+^(*C*_*3v*_)­^a^

neutral complex	**Δ** **^2^ *E*(*AB*)**	*E*_*R*_(B_2_H_4_)	*E*_*R*_(N-base)	Δ*E*
B_2_H_4_–guanidine (**A**)	–340.4	142.8	22.9	–174.7
B_2_H_4_–guanidine (**B**)	–302.6	127.6	11.8	–163.2
B_2_H_4_–1-methyl-imidazole	–270.0	116.7	5.6	–147.7
protonated complex				
B_2_H_5_–guanidine]^+^ (**A**)	–657.0	219.4	37.7	–400.0
[B_2_H_5_–guanidine]^+^ (**B**)	–651.5	206.0	36.8	–408.7
[B_2_H_5_–1-methyl-imidazole]^+^	–585.7	193.6	12.0	–380.0

a All values are in kJ·mol^–1^.

Focusing now on 1-methylimidazole, we find that the stabilizing Δ^2^ *E*(*AB*) term is consistently about 70 kJ·mol^–1^ larger for guanidine than for 1-methylimidazole. Upon protonation, however, the most stable guanidine isomer corresponds to form **B**, which slightly reduces this difference to approximately 66 kJ·mol^–1^ for the protonated species. This effect is partially counterbalanced by larger monomer distortion energies for guanidine, which are again more destabilizing for the neutral species (ca. 43 kJ mol^–1^) than for the protonated ones (ca. 37 kJ·mol^–1^). Therefore, the enhanced stability of the neutral guanidine complex relative to the neutral 1-methylimidazole complex differs in only 2 kJ·mol^–1^ from that observed for the corresponding protonated species (27 vs 29 kJ·mol^–1^). Since the stabilization of the protonated complex relative to the neutral species provides a measure of the basicity enhancement (see eq 4 and Figure 6), the difference in basicity enhancement between 1-methylimidazole and guanidine amounts to only ∼2 kJ·mol^–1^, despite guanidine being intrinsically the stronger base. A graphic representation of this far from trivial energy balance is provided in Figure 8, which shows that the binding enthalpies of guanidine with B_2_H_4_ and B_2_H_5_^+^ (−166.3 and −391.9 kJ·mol^–1^, respectively) are 23.7 and 21.6 kJ·mol^–1^ larger, respectively, than those of 1-methylimidazole (−142.6 and −370.2 kJ·mol^–1^, respectively). Obviously, if these two differences (23.7 and 21.6 kJ·mol^–1^) were identical, the PAs of the two complexes would be the same. However, because the enthalpy increase upon protonation is 2 kJ·mol^–1^ smaller for the guanidine complex than for the 1-methylimidazole complex, the PA of B_2_H_4_-guanidine must be lower by the same amount, despite guanidine being the stronger base. A similar analysis could be done for the other methylimidazole derivatives (see Table S1).

**Figure 8:**
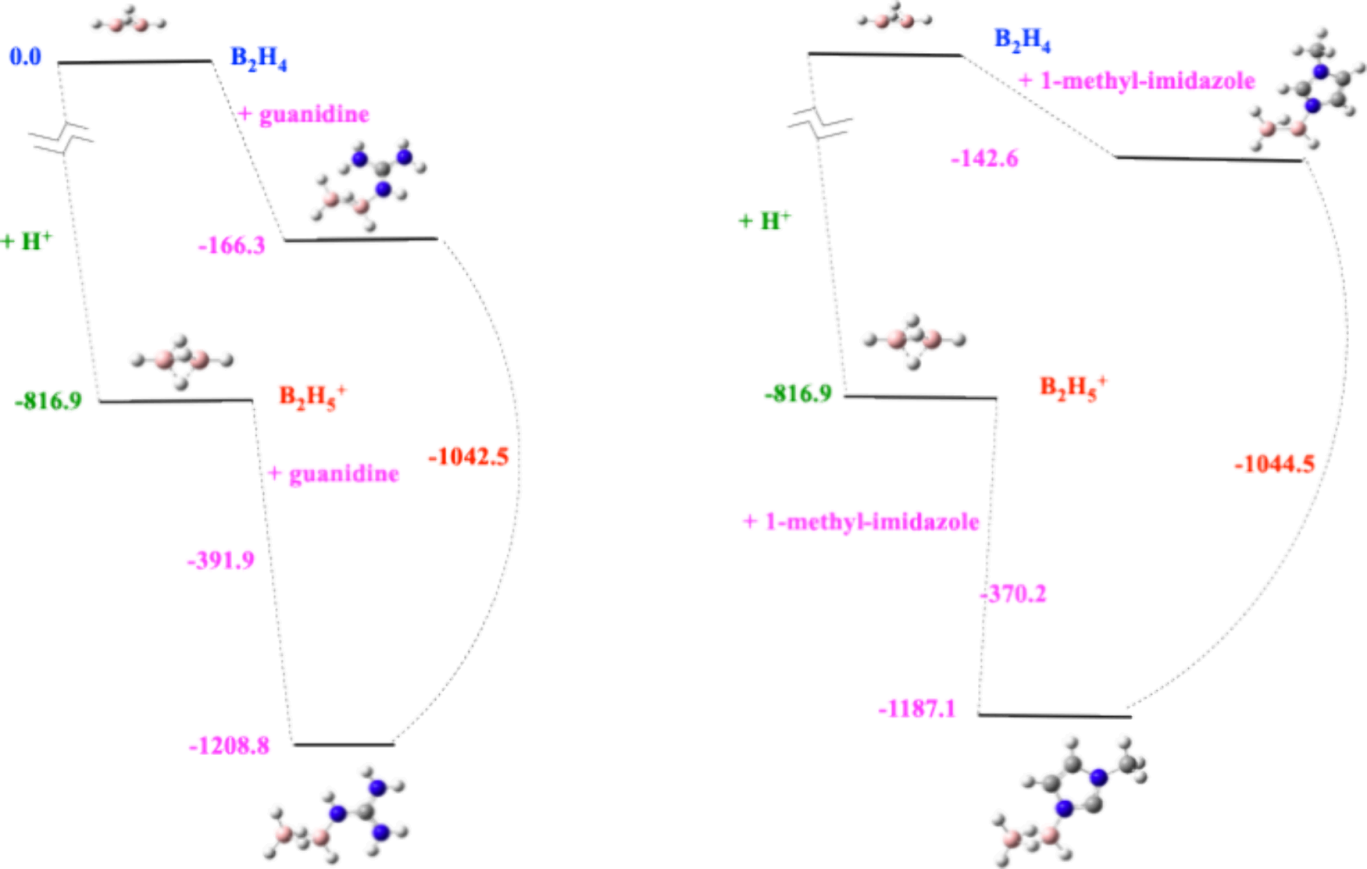
Diagram illustrating two alternative paths connecting B_2_H_4_ with complexes in which its protonated form interacts with guanidine and 1-methylimidazole. The diagram highlights the origins of the different basicity enhancements induced by these two nitrogen bases on the basicity of B_2_H_4_. G4-enthalpy values are in kJ·mol^–1^.

## Conclusions

The bonding peculiarities of the *C*_*2v*_ isomer of B_2_H_4_ are behind its electron donor capacity. In this study, we demonstrate that the interaction of this isomer with various nitrogen bases not only enhances its stability—so much so that, for pyridine and stronger nitrogen bases, the complexes derived from this C_2v_ form are the only stable ones—but also leads to a dramatic increase in its intrinsic basicity. Among the different protonation sites, boron is the most basic site and far more basic than nitrogen. Consequently, the protonated complexes can be viewed as the association of the different nitrogen bases to the B_2_H_5_^+^ cation. This viewpoint allowed us to show that the intrinsic basicity of B_2_H_4_ increases drastically upon association with all nitrogen bases studied. Indeed, the B_2_H_4_–pyridine complex falls within the range of gas-phase superbases, with a calculated proton affinity (PA) exceeding 1000 kJ·mol^–1^.^36^ Complexes formed with bases stronger than pyridine, such as guanidine and methyl-substituted imidazoles, display even greater basicity, surpassing that of the prototypical superbase 1,8-bis­(dimethylamino)­naphthalene (proton sponge).^35^ Notably, the B_2_H_4_–1,2,5-trimethylimidazole complex is predicted to be 34 kJ·mol^–1^ more basic than the proton sponge, corresponding to an increase in the protonation equilibrium constant of nearly 6 orders of magnitude.^37^

It is worth noting that, very recently, we have shown that certain compounds, that can be viewed as derivatives of B_2_H_4_, such as the diborane derivatives of dipyrazole, also exhibit PAs larger than that of the proton sponge.^35^ In the latter case, the exceptionally high basicity arises from the release of molecular strain on going from the neutral to the protonated form. A final remark: in all these proton sponges, the basic site is a prototypic electrodeficient atom, boron.

## Supplementary Material



## Notes

Description of the nitrogen and hydride protonation processes, the structures of the complexes formed between B_2_H_4_ and the protonated N-Bases, the molecular graph for the B_2_H_4_–NH_4_^+^ and the [B_2_H_3_–NH_3_ ]^+^···H_2_ complexes, the thermodynamic cycles for the hydride abstraction and the N-protonation processes for the B_2_H_4_–NH_3_ and B_2_H_4_-guanidine complexes, an alternative view of the molecular electrostatic potential of B_2_H_4_ showing their maxima associated with the hydrogen atoms, and the G4 MBIE results for B_2_H_4_–N-Base and [B_2_H_5_–N-Base]^+^ complexes (PDF)
