# Supplementary material for: Electrodeficient Diborane (4) Converted into an Extraordinary Proton Sponge
Source: J Phys Chem A. 2026 Jan 29;130(6):1330–7. doi: 10.1021/acs.jpca.5c08104 (PMC12908114; doi:10.1021/acs.jpca.5c08104)
Supplement: Supplementary file 1 [file jp5c08104_si_001.pdf]

Electrodeficient Diborane (4) Converted into an Extraordinary Proton Sponge

Manuel Yáñez[1,*], M. Merced Montero-Campillo[1], Otilia Mó[1], and Ibon Alkorta[2,*]

[1] Departamento de Química, Módulo 13, Facultad de Ciencias, and Institute for Advanced Research in Chemical Sciences (IAdChem), Universidad Autónoma de Madrid, Campus de Excelencia UAM-CSIC, Cantoblanco, 28049 Madrid, Spain.

[2] Instituto de Química Médica, IQM-CSIC, Juan de la Cierva, 3. 28006 Madrid, Spain.

* manuel.yanez@uam.es, ibon@iqm.csic.es

Supporting Information

CONTENTS

Addendum. Nitrogen and hydride protonation processes.

Figure S1 Structures of the complexes formed between B_2H_4 and the protonated N-bases.

Figure S2. Molecular graph for the B_2H_4-NH_4^+ complex.

Figure S3. Molecular graph for the $[B_2H_3\text{-}NH_3]^+\cdots H_2$ complex.

Figure S4. Thermodynamic cycles for the hydride abstraction and the N-protonation processes for the B_2H_4–NH_3 and B_2H_4-guanidine complexes.

Figure S5. Alternative view of the molecular electrostatic potential of B_2H_4 showing their maxima associated with the hydrogen atoms.

Table S1. G4 MBIE results for B_2H_4–N-base and $[B_2H_5\text{–N-base}]^+$ complexes.

Addendum. Nitrogen and hydride protonation processes.

As indicated in the main text, protonation of the complexes formed between B_2H_4 and nitrogen bases can in principle occur at the hydride sites (i.e., the hydrogen atoms bound to boron) or at the nitrogen atom of the base. However, neither of these pathways can compete with protonation at the boron atom. The complexes in which the protonation takes place at the N-base are systematically stabilized by the formation of a hydrogen bond between the protonated base and the B–B bond of the B_2H_4 moiety (see Figure S1).

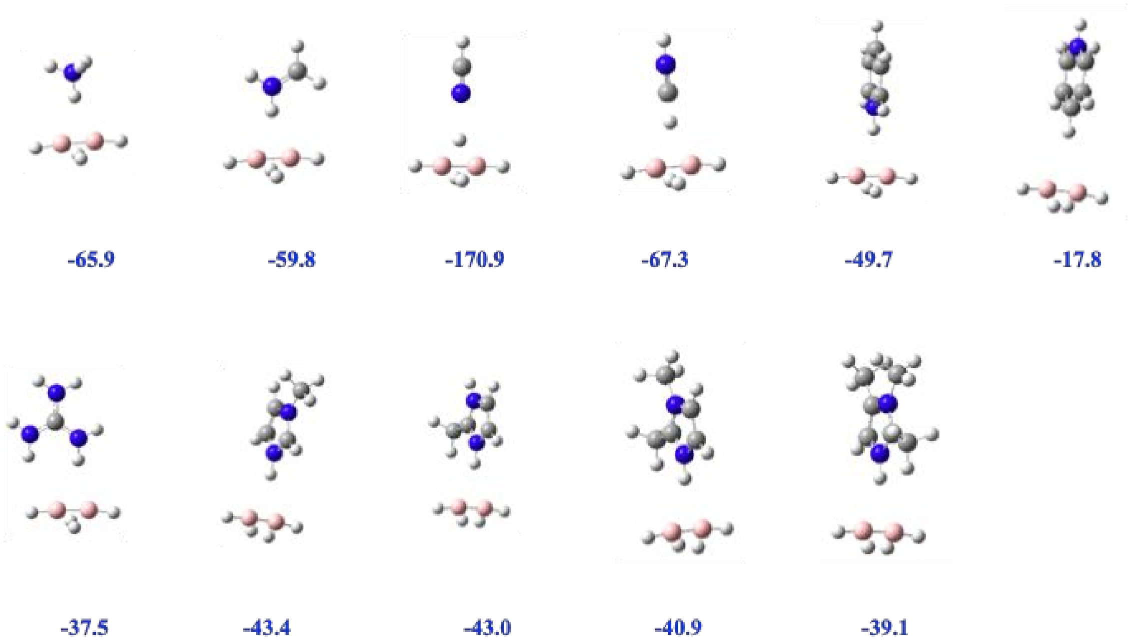

Figure S1 Structures of the complexes formed between B_2H_4 and the protonated N-bases. Values correspond to their dissociation enthalpies (kJ·mol^{-1}) into B_2H_4 + N-baseH^+.

Accordingly, these complexes are characterized by the presence of a BCP between the hydrogen atom of the base and the NNA at the middle of the B-B (see Figure S2 for the case of the complex with NH_4^+).

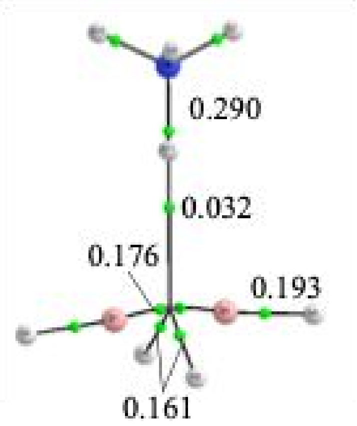

Figure S2. Molecular graph for the B_2H_4–NH_4^+ complex. Electron densities at the BCPs (green dots) are in a.u.

The second set of protonated species, those arising from the protonation at the hydride atoms, are systematically a complex between the H_2 molecule and the $[B_2H_3\text{-N-base}]^+$ cation (see Figure S3). In all cases the most stable complex corresponds to than in which the hydride abstraction process involves de BH_2 group that does not interact with the base.

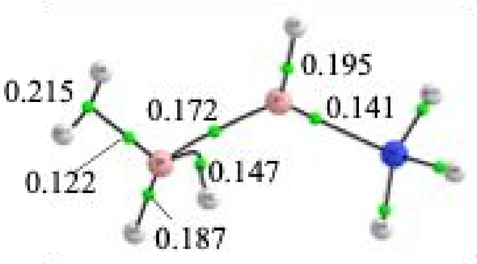

Figure S3. Molecular graph for the $[B_2H_3\text{-}NH_3]^+\cdots H_2$ complex. Electron densities at the BCPs (green dots) are in a.u.

When the relative stabilities of these two alternative protonated species are compared, it is striking that protonation at the nitrogen atom of the base is less favorable than protonation at the hydride centers. This trend can be rationalized by considering two key factors. First, protonation at a hydride center corresponds to a hydride abstraction process, leading to the formation of H_2, which subsequently forms a weakly bound complex with the remaining $B_2H_3^+$–N-base fragment. This highlights the importance of the hydride donor ability of the

complex: a lower endothermicity associated with H^- loss favors the hydride abstraction pathway. Second, the high exothermicity of H_2 formation from protonation of H^- further stabilizes the process. At the G4 level of theory, the enthalpy of this reaction is calculated to be −1712.2 kJ·mol^{-1}. Both factors together favor, as illustrated in Figure S4 for the specific cases of NH_3 and guanidine complexes, the hydride abstraction process over N-base protonation. Indeed, although moving from NH_3 to guanidine leads to a significant increase in basicity –thus enhancing the exothermicity of protonation at the nitrogen center– this effect is mitigated by the higher dissociation enthalpy of the B–N bond. At the same time, guanidine exhibits a greater hydride-donor ability than ammonia, making hydride loss a less endothermic process. Altogether, these factors explain why, for ammonia, hydride abstraction is approximately 19 kJ·mol^{-1} more exothermic than protonation at the nitrogen atom, whereas for guanidine this difference increases to about 80 kJ·mol^{-1}.

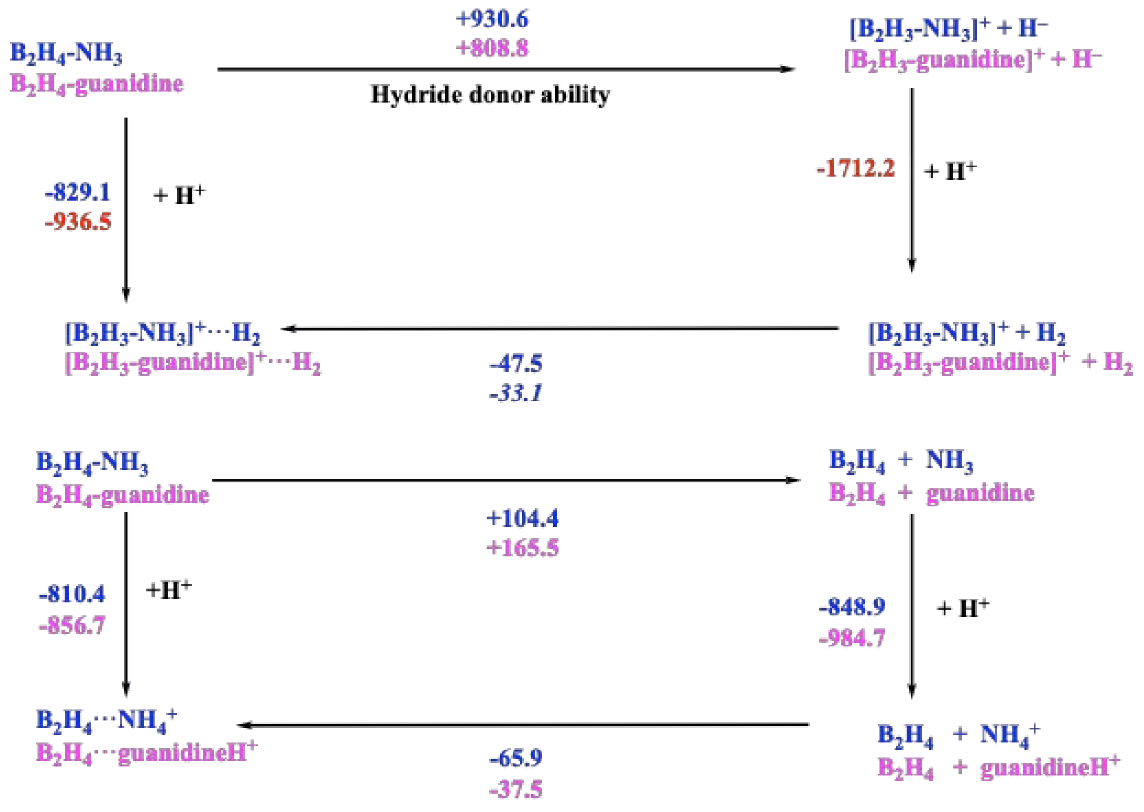

Figure S4. Thermodynamic cycles for the hydride abstraction and the N-protonation processes for complexes of the B_2H_4–NH_3 (values in blue) and B_2H_4-guanidine (values in magenta) complexes. The value in red corresponds to the enthalpy of the reaction in which H_2 is formed via protonation of H^-. All values are in kJ·mol^{-1}.

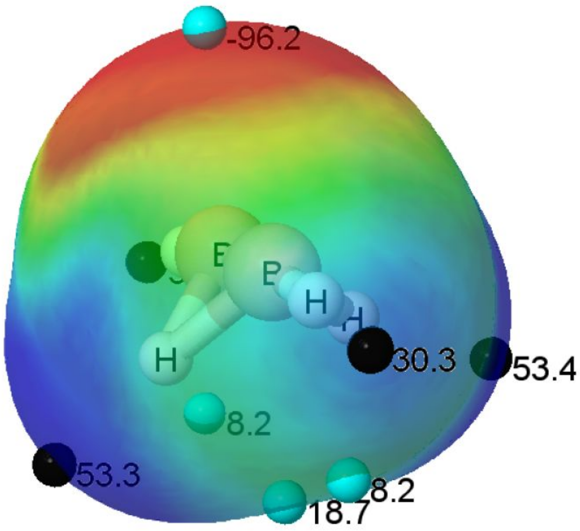

Figure S5. Alternative view of the molecular electrostatic potential of B_2H_4 showing their maxima (in kJ·mol^{-1}) associated with the hydrogen atoms. Red area is the nucleophilic region.

Table S1. G4 MBIE results for B_2H_4–N-base and $[B_2H_5$–N-base$]^+$ complexes formed by attaching the N-base to the most stable conformers of B_2H_4 (C_{2v}) and $B_2H_5^+$(C_{3v}). All values are in kJ·mol^{-1}.

	$\Delta^2E(AB)$	$E_R(B_2H_4)$	E_R(N-base)	ΔE
Neutral complexes				
B_2H_4–NH_3	-217.4	105.4	0.5	-111.5
B_2H_4–NH=CH_2	-216.9	126.4	2.9	-140.5
B_2H_4–NCH	-178.4	110.0	0.1	-68.2
B_2H_4–pyridine	-264.0	124.1	3.0	-139.9
B_2H_4–1-methyl-imidazole	-270.0	122.3	2.9	-147.7
B_2H_4–2-methyl-imidazole	-277.7	128.5		-149.3
B_2H_4–1,2-dimethyl-imidazole	-281.8	129.1	0.3	-152.8
B_2H_4–1,2,5-trimethyl-imidazole	-286.0	130.3	2.4	-155.7
Protonated complexes				
$[B_2H_5$–$NH_3]^+$	-440.9	175.8	0.8	-264.4
$[B_2H_5$–pyridine$]^+$	-533.4	193.8	5.8	-333.7
$[B_2H_5$–1-methyl-imidazole$]^+$	-585.7	197.4	12.0	-380.2
$[B_2H_5$–2-methyl-imidazole$]^+$	-577.8	206.0	14.4	-380.4
$[B_2H_5$–1,2-dimethyl-imidazole$]^+$	-610.8	199.6	17.3	-393.9
$[B_2H_5$–1,2,5-trimethyl-imidazole$]^+$	-626.8	200.8	19.2	-406.7
